# An effective detachment system for human induced pluripotent stem cells cultured on multilayered cultivation substrates using resonance vibrations

**DOI:** 10.1038/s41598-019-51944-w

**Published:** 2019-10-30

**Authors:** Yusuke Terao, Yuta Kurashina, Shugo Tohyama, Yuki Fukuma, Keiichi Fukuda, Jun Fujita, Kenjiro Takemura

**Affiliations:** 1 School of Science for Open and Environmental Systems, Graduate School of Science and Technology, Keio University, 3-14-1 Hiyoshi, Kohoku-ku, Yokohama 223-8522 Japan; 2 Department of Mechanical Engineering, Faculty of Science and Technology, Keio University, 3-14-1 Hiyoshi, Kohoku-ku, Yokohama 223-8522 Japan; 3 Department of Materials Science and Engineering, School of Materials and Chemical Technology, Tokyo Institute of Technology, 4259 Nagatsutacho, Midori-ku, Yokohama 226-8503 Japan; 4 Department of Cardiology, Keio University School of Medicine, 35 Shinanomachi, Shinjuku-ku, Tokyo 160-8582 Japan; 5 Department of Organ Fabrication, Keio University School of Medicine, 35 Shinanomachi, Shinjuku-ku, Tokyo 160-8582 Japan

**Keywords:** Biophysical methods, Biophysical methods, Induced pluripotent stem cells, Induced pluripotent stem cells, Biomedical engineering

## Abstract

Clinical application of human induced pluripotent stem cells (hiPSCs) has been hampered by the lack of a practical, scalable culture system. Stacked culture plates (SCPs) have recently attracted attention. However, final cell yields depend on the efficiency of cell detachment, and inefficient cell recovery from SCPs presents a major challenge to their use. We have developed an effective detachment method using resonance vibrations (RVs) of substrates with sweeping driving frequency. By exciting RVs that have 1–3 antinodes with ultra-low-density enzyme spread on each substrate of SCPs, 87.8% of hiPSCs were successfully detached from a 5-layer SCP compared to 30.8% detached by the conventional enzymatic method. hiPSC viability was similar after either method. Moreover, hiPSCs detached by the RV method maintained their undifferentiated state. Additionally, hiPSCs after long-term culture (10 passages) kept excellent detachment efficiency, had the normal karyotypes, and maintained the undifferentiated state and pluripotency. These results indicated that the RV method has definite advantages over the conventional enzymatic method in the scalable culture of hiPSCs using SCPs.

## Introduction

Human induced pluripotent stem cells (hiPSCs) have the potential to differentiate into any type of cell, and can be used for disease modeling and drug discovery. They have also shown promise as a cell source for regenerative therapies^1–4^. In 2014, hiPSC-derived retinal pigment epithelial cell sheets were generated for treatment of age-related macular degeneration, and the cells were successfully transplanted for the first time by Takahashi *et al*.^5^. However, the number of transplanted cells was relatively small (graft size: 1.3 × 3 mm^2^, cell density: 4,500 < viable cells/mm^2^ < 29,000). On the basis of the success of the first clinical trial, hiPSCs have also received broad attention in regenerative therapies for much larger organs, such as the heart, liver, and spine^6–8^. Notably, heart regenerative therapies require a large number of cultured cells, because the heart consists of 1 × 10^9^–10^10^ cardiomyocytes^9^. To prepare such a large number of high-quality cardiomyocytes, many hiPSCs capable of cardiac differentiation are required, because terminally differentiated cardiomyocytes lose their proliferation ability^10^.

Several scalable hiPSC culture techniques have been investigated^11^, but highly efficient and stable mass culture is still in the trial phase for clinical study. Three-dimensional (3D) suspension culture, 3D sphere culture^12^, microcarrier suspension culture^13,14^, and spinner and bioreactor culture methods^15–17^ have been researched. On the other hand, even though 2D culture systems have several advantages for hiPSC culture, especially in the maintenance of pluripotency and quality control, they are thought inappropriate for mass culture, because the limited culture surface restricts maximum cell numbers and demands intensive labor.

As for an efficient method for cell detachment, we detached cells from small-scale cell culture dishes^18–20^ using resonance vibration (RV). Employing RV of dishes enables us to effectively obtain cells without damage compared to enzyme detachment methods. As for the long-term culture, the automated 2D hiPSCs culture system on the dish was developed and the obtained hiPSCs had a potency of differentiation into three germ layer cells^21^. The dexterous motion of robot arms mimicking manual procedures makes it possible to automate the culture process. The massive scale culture system must be invented to apply cell therapies to large organs, as well. 10^8^–10^9^ cells (30–300 ø60 culture dishes) are required, for instance, for the heart regeneration medicine. To overcome the drawbacks of 2D culture systems, we established a mass-2D culture system for hiPSCs using stacked culture plates (SCPs) and active gas ventilation^22^.

Although these scalable culture systems enable large-scale cultivation of hiPSCs, both 3D and 2D culture systems require efficient cell recovery ratios to be useful. However, SCPs were not originally designed for recovery of adhesive cells, but for collection of supernatants to obtain vaccines and antibodies^23,24^; thus, the process of detaching cells from SCPs is challenging. The chief difficulties are the limited mechanical stimulus provided by manual tapping in the detachment process and inefficient enzymatic activity. The total weight of a 10-layer SCP including culture medium is approximately 2 kg. Due to such a heavy weight, cell detachment efficiency highly depends on manual technique. Moreover, it is difficult to evenly spread the detaching solutions in all layers of multilayer systems.

To improve the cell collection ratio, mechanical stimuli should be applied equally and efficiently to all layers of the SCP system. In this study, we propose a new, efficient, and safe method of detaching large numbers of hiPSCs from all layers of an SCP system using RVs, which will contribute to the clinical and industrial application of hiPSCs. That is, we are able to apply sufficient stimulus to cells with a small excitation force, and detach cells efficiently by using RVs on the SCPs.

## Results

### Cell detachment system and vibration characteristics

**Figure 1:**
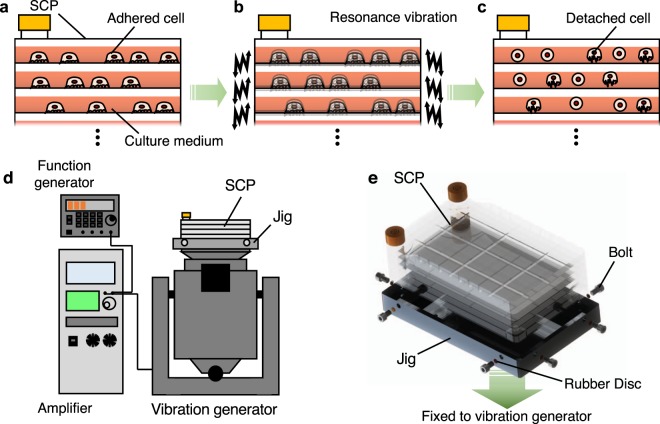
Concept of the cell collection process using resonance vibrations and the vibration excitation system developed to excite resonance vibrations on the cultivation layers of SCPs. (**a**) Cells adhere to each cultivation layer. **(b)** Resonance vibrations are excited on each layer. **(c)** Cells are detached from each layer. Whole composition of **(d)** the vibration excitation system and **(e)** the method of fixing the SCP to the jig, which is bolted to the vibration generator. The SCP is put into the aluminum jig and fixed by bolts. Rubber discs are placed in between the bolts and SCP.

The cell detachment system developed in this study is schematically illustrated in Fig. 1. Cells are cultured on each substrate layer of an SCP (Fig. 1a) and are subjected to a trypsin solution and RVs induced in each substrate layer by a vibrator (Fig. 1b); this combination of trypsin and vibration detaches the cells (Fig. 1c). The system as shown in Fig. 1d is composed of an SCP, a vibration generator and a jig used to fix the SCP onto the vibration generator. The SCP is mounted on the aluminum and fixed by bolts as shown in Fig. 1e. Rubber discs are placed in between the bolts and SCP. Note that the jig is bolted to the stage of the vibration generator. A swept frequency input signal was applied to the vibration generator using a function generator (WF1946B; NF Corporation, Yokohama, Japan) and an amplifier (HSA4011; NF).

**Figure 2:**
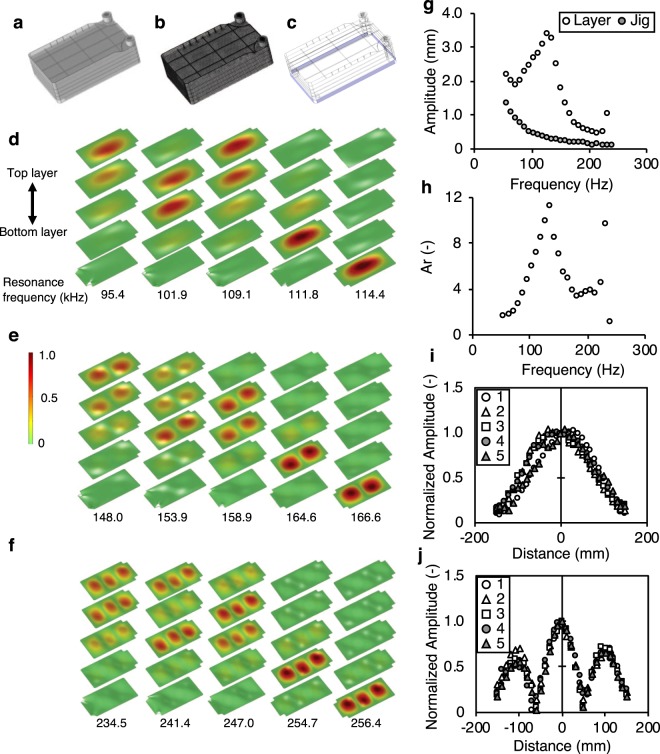
Three-dimensional (3D) CAD model of a 5-layer SCP used for eigenvalue analysis, results of eigenvalue analysis, and measurement by laser Doppler velocimetry. (**a**) 3D CAD model, **(b)** 3D mesh model using tetrahedral elements; **(c)** side planes colored purple are fixed for eigenvalue analysis. **(d)** The eigenvalue analysis of first out-of-plane resonant mode of SCP. Resonance frequency ranges between 95.4 and 114.4 Hz. **(e)** The eigenvalue analysis of second out-of-plane resonant mode of SCP Resonance frequency ranges between 148.0 and 166.6 Hz. **(f)** The eigenvalue analysis of third out-of-plane resonant mode of SCP. Resonance frequency ranges between 234.5 and 256.4 Hz. **(g)** Vibration amplitude at the center of the cultivation layer and the jig and **(h)** relative amplitude, *A*_r_. The distributions of vibration amplitude are measured along the longer central axis of each cultivation layer with an input frequency corresponding to each resonance frequency of **(i)** the first out-of-plane resonant mode and **(j)** third out-of-plane resonant mode listed in Table SI. These amplitudes are normalized to their maximum values.

To accurately apply vibration stimuli to the cells on each substrate, the first to third out-of-plane RVs of each substrate of a single-layer SCP (Fig. S1) and 5-layer SCP (Fig. 2) were excited. The resonance frequency for each vibration mode was confirmed in advance by eigenvalue analysis using finite element analysis software (COMSOL Multiphysics 5.2, COMSOL Inc., Burlington, MA, USA). As shown in Fig. 2d–f, the resonance frequency of each vibration mode for each layer is slightly different due to the boundary conditions of each layer. Consequently, the exciting frequency was swept between 50 Hz and 300 Hz over 30 s to apply vibration stimuli to the cells.

The vibration characteristics of the SCP were evaluated. Figure 2g shows vibration amplitudes at the center of the upper substrate of the 5-layer SCP and at the jig. Since the input power to the vibration generator is constant, the vibration amplitude of the jig lowers as the input frequency increases. Figure 2h shows the relative amplitude, *A*_*r*_, of the upper substrate against that of the jig. Resonance frequencies are defined at 134 Hz and 230 Hz for the first and third RVs. Note that the second resonance should exist between the first and third resonance; however, it cannot be monitored because the measuring point is located at the center of the substrate where the node for the second resonance mode is. Figure 2i,j show the amplitude distributions of the first and the third RVs, respectively. Note that vibration amplitudes are normalized to their maximum values. The first and third out-of-plane RVs are excited when the exciting frequency corresponds to each resonance frequency summarized in Table S1. The resonance frequencies for the first and the third vibration modes are located between 114 and 134 Hz and between 222 and 246 Hz, respectively.

### Cell detachment experiments with mouse myoblast cells

**Figure 3:**
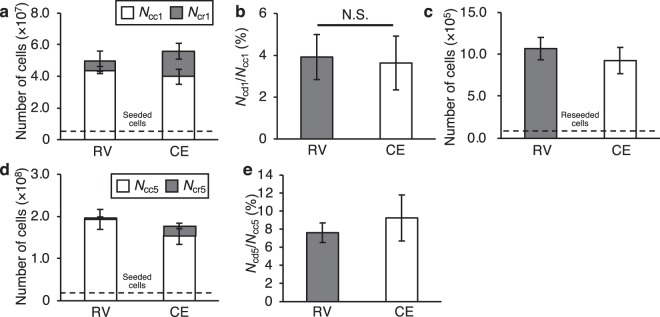
Results of cell detachment experiment employing C2C12 mouse myoblast cells. (**a**–**c**) Experiments employing single-layer SCPs. (**a**,**b**) The number of seeded cells was 3.5 × 10^6^ (mean ± SD, *n* = 5). Cells were cultured for 4 days, then collected by the resonance vibration (RV) method and the conventional enzymatic (CE) method and measured the number of cells by hemocytometer. Vibration was excited for 1 min after trypsin treatment for 2 min. The sweep frequency range was from 50 to 250 Hz and sweep cycle was 30 s. **(a)** The number of cells collected, *N*_cc1_, and remaining, *N*_cr1_, after each method. **(b)** The percentage of dead cells (*N*_cd1_/*N*_cc1_) obtained by dividing the number of dead cells, *N*_cd1_, by the number of collected cells, *N*_cc1_. **(c)** The number of cells obtained by reseeding cells detached by the RV and CE methods into 60 mm dishes followed by culture for 72 h. The number of reseeded cells, *N*_cs1_, was 8.0 × 10^4^. **(d**,**e)** Experiments employing 5-layer SCPs. The number of seeded cells is 1.75 × 10^7^ (mean ± SD, *n* = 4). Cells were cultured for 4 days, then collected by the RV and CE methods. Vibration was excited for 1 min after trypsin treatment for 2 min and measured the number of cells by hemocytometer. The sweep frequency range was from 50 to 300 Hz and the sweep cycle was 30 s. **(d)** The number of cells collected, *N*_cc5_, and remaining, *N*_cr5_, after each method. **(e)** The percentage of dead cells (*N*_cd5_/ *N*_cc5_) obtained by dividing number of dead cells, *N*_cd5_, by the number of collected cells, *N*_cc5_.

To examine the efficacy of the proposed cell detachment method using RVs, we evaluated the number of live/dead cells, viability, and proliferation after detachment using the mouse myoblast cell line C2C12 (RCB0987; Riken Bio Resource Center, Ibaraki, Japan). We chose myoblasts because they are typical cells studied in tissue engineering and regenerative medicine, such as in regeneration models of skeletal muscle tissue^25^ and myoblast sheets for improving cardiac function^26^. The appropriate sweep frequency and trypsinization time are defined in the Experimental Procedures. Figure 3a–c show the results of the experiment employing a single-layer SCP, and Fig. 3d,e show the results employing a 5-layer SCP. Cells were cultured for 4 days in advance and collected by the RV method. As a control, a technician collected cells with a typical trypsinization and hand taps (denoted the conventional method). Figure 3a shows the number of cells collected, *N*_cc1_, and remaining, *N*_cr1_, after collection by each method. Overall, 88.8% and 71.1% of the total cells (*N*_cc1_ + *N*_cr1_) were detached (*p* < 0.05, Student’s t-test) by the RV and the conventional enzymatic (CE) methods, respectively. Figure 3b shows the percentage of dead cells, *N*_cd1_, divided by *N*_cc1_. With each method, 3.6% and 3.9% of *N*_cc1_ were dead. To evaluate proliferation ability after detachment, cells (*N*_cp1_) detached by the RV and the CE methods were reseeded into 60 mm dishes, cultured for 72 h, and counted (Fig. 3c). *N*_cp1_ values after the RV and the CE methods were 10.7 × 10^5^ and 9.3 × 10^5^, respectively. Figure 3d shows the number of cells collected, *N*_cc5_, and remaining, *N*_cr5_, by each method from a 5-layer SCP. As shown, 98.4% and 87.2% of the cells were detached (*p* < 0.01, Student’s t-test) by the RV and CE methods, respectively. Figure 3e shows the percentage of dead cells, *N*_cd5_, divided by *N*_cc5_, and 7.6% and 9.3% of *N*_cc5_ were dead after collection by the RV and CE methods, respectively.

### Cell detachment experiments with hiPSCs

**Figure 4:**
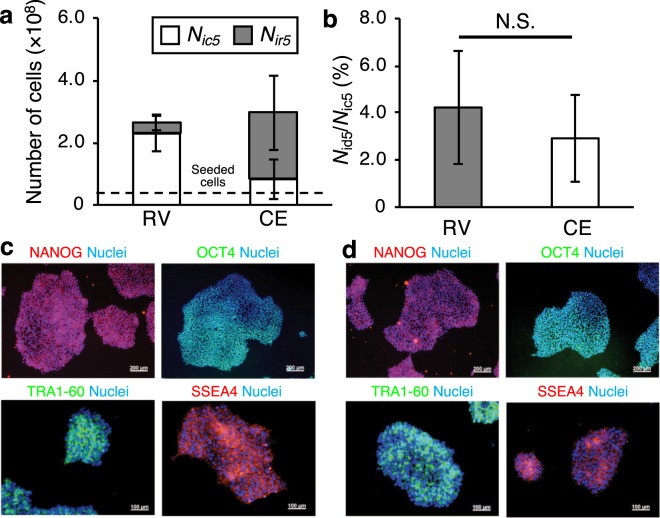
Results of cell detachment experiment employing hiPSCs. (**a**,**b)** Experiments using 5-layer SCPs. The number of seeded cells was 4.0 × 10^7^ (mean ± SD, *n* = 4). Cells were cultured for 4 days, collected by the resonance vibration (RV) method and the conventional enzymatic (CE) method and measured the number of cells by hemocytometer. Vibration was excited for 1 min after TrypLE Select treatment for 2 min. The sweep frequency range was from 50 to 300 Hz and sweep cycle was 30 s. **(a)** The number of cells collected, *N*_ic5_, and remaining, *N*_ir5_, after each method. **(b)** The percentage of dead cells (*N*_id5_/ *N*_ic5_) obtained by dividing number of dead cells, *N*_id5_, by the number of collected cells, *N*_ic5_. **(c**,**d)** Representative immunofluorescence staining for NANOG (red), OCT4 (green), TRA1-60 (green), SSEA4 (red), and nuclei (blue) in hiPSC-derived dispersed cells detached by **(c)** the RV and **(d)** the CE methods. Scale bars represent 200 µm (NANOG and OCT4) and 100 µm (TRA1-60 and SSEA4).

To validate the efficacy of the RV method for culturing PSCs, we conducted a similar experiment using hiPSCs (253G4; Kyoto University, Kyoto, Japan). We chose hiPSCs because they are typical stem cells that can be applied in tissue engineering and regenerative medicine. After detachment, we evaluated the representative immunofluorescence staining for NANOG, OCT4, TRA1-60, SSEA4, and nuclei in hiPSC-derived dispersed cells to validate the undifferentiated state of the cells collected with the RV method. Figure 4 shows the results of the experiments employing a 5-layer SCP. Figure 4a shows the number of cells collected, *N*_ic5_, and remaining, *N*_ir5_, by each method. Overall, 87.8% and 30.8% of the total cells (*N*_ic5_ + *N*_ir5_) were detached (*p* < 0.01, Student’s t-test) by the RV and CE methods, respectively. Figure 4b shows the percentage of dead cells, *N*_id5_, divided by *N*_ic5_. There was no significant difference in the percentage of dead *N*_ic5_ cells after collection by either method. Furthermore, the morphologies of hiPSCs detached by each method were normal, and the cells were clearly stained with NANOG, OCT4, TRA1-60, and SSEA4, as shown in Fig. 4c,d.

### Cell detachment during 10 passages with hiPSCs

**Figure 5:**
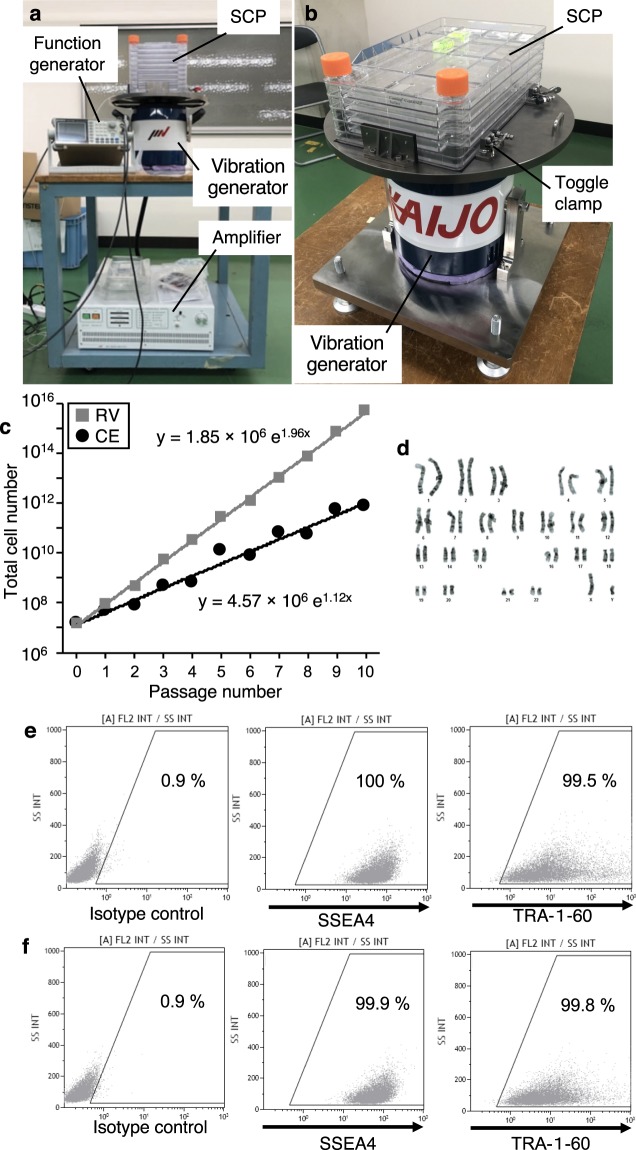
Long term culture of 10 passages with hiPSCs using resonance vibrations. **(a**,**b)** The optimal vibration extension system for long term culture. Whole composition of the vibration excitation system **(a)**, and installation of the SCPs mounted on the vibration system on the ground **(b)**. **(c)** The results of the growth curve during 10 passages collected by the resonance vibration (RV) method and the conventional enzymatic (CE) method. **(d)** The karyotypes of the cultured hiPSCs were observed. **(e,f)** Representative flow cytometory analysys (FCA) for SSEA4 and TRA1-60 positive cells passaged by **(e)** the RV method and **(f)** the CE method. Photographs (**a**,**b**) permission acquired from SHIBUYA Corporation.

In order to evaluate the safety of hiPCS using our method, we show the growth curve during 10 passages, karyotypes, representative immunofluorescence staining and differentiation state after 10 passages. The total number of cells detached by RV is greater than those detached by CE from the results of the growth curve during 10 passages as shown in Fig. 5a. Hence, the result indicates that the growth curve of RV method increased greatly compared to that of the CE method as the number of passages increased. To confirm the safety of the chromosomes after repeated passages with RV, the karyotypes were observed (Fig. 5b). The karyotypes of the cultured hiPSCs was normal. In order to evaluate the undifferentiation state and pluripotency of the passaged cells, immunofluorescence staining and the flow cytometry analysis (FCA) were performed. Immunofluorescence staining confirmed strong expression of pluripotency markers in all colonies (Supplementary Fig. 3a,b). The FCA confirmed that following this metabolic selection, almost all cells (>99.5%) clearly expressed SSEA4 and TRA-1-60 (Fig. 5c,d). These data strongly indicated that the cells passaged by the RV method remained undifferentiated. Additionally, ectoderm, mesoderm, and endoderm differentiated from hiPSCs passaged by RV method were shown in Fig. 5e for the maintenance of pluripotency in hiPSCs.

## Discussion

After evaluating the vibration characteristics of the developed cell detachment system, we conducted detachment experiments with the mouse myoblast cell line C2C12 on single-layer and 5-layer SCPs to evaluate detachment efficiency, viability, and proliferation of the detached cells. Our results indicate that cells were detached more effectively by the RV method mediated by RVs of substrates than by the CE method of trypsinization. Additionally, viability and proliferation of the detached cells were similar after both detachment methods. Moreover, we conducted similar experiments using hiPSCs and validated the undifferentiated state of the detached cells by representative immunofluorescence staining analyses. The results indicate that the RV method is applicable to SCPs and maintains cell viability and the undifferentiated state.

The resonance frequencies of the employed vibration modes of each layer of an SCP are different, as shown in Table S1 and Fig. 2d–f. This difference is mainly caused by the constraint conditions of each layer. The bottom layer, for example, has four layers above it and is fixed to the jig around its circumference. On the other hand, the top layer has four layers below it. Owing to this difference in resonance, swept frequencies are required to excite the first, second, and third RVs on all layers. In this way, we can excite multiple RV modes of each layer, resulting in the application of mechanical stimuli to a wide area of each layer. That is, we can eliminate the presence of a vibration node from each resonance mode. In addition, sweeping the driving frequency has the advantage of exciting resonance vibrations with certainty. Generally, the resonance frequency of any elastic body is unstable depending on the, constraint conditions, and temperature, among other things. In the case of an SCP, its resonance frequency may depend on the volume of inner solution, surrounding temperature, and fixing conditions. Sweeping the input frequency overcomes this instability of resonance frequency and makes the RV method robust.

Equations S4 and S6 in the SI prove that the force applied to the cells is 10–1000 times higher during detachment by the RV method than that applied by the CE method. Moreover, in the CE method, the force is only applied once and briefly by a single tap. On the other hand, in the RV method, the force is continuously applied during excitation of the vibrations. The above estimation of the force applied to the cells suggests that the cell detaching efficiency can be improved with the RV method due to the significant improvement of the force applied to the cells.

In the detachment process, we used the enzymes trypsin-EDTA for C2C12 and TrypLE Select for hiPSCs. These proteinases may damage cell surfaces^27,28^; therefore, enzyme concentration and treatment duration are important factors, because the opportunity for the enzyme to break down cell surface proteins increases with both^29,30^. For this reason, the enzymes were diluted to 0.0125% trypsin-EDTA and 50% TrypLE Select, which are half the typical concentrations, and the durations of treatment were defined as 3 min and 5 min, which are the minimum times required for general immersion. The experimental results using the 5-layer SCP indicate that the percentage of total cells collected was approximately 90% after collection by the RV method, improving more than 10% and 50% for C2C12 cells and hiPSCs, respectively, compared with the percentage collected by the CE method, as shown in Figs 3d and 4a. This demonstrates the remarkable effectiveness of the RV cell detachment method.

The differences between the detachment ratios of C2C12 cells and hiPSCs may be due to differences in the protein degradation ability of trypsin-EDTA and TrypLE Select. Regardless, the RV method improves the detachment efficiency of both C2C12 cells and hiPSCs. This improvement will be more marked in larger-scale cell cultivation. The RV method also showed excellent detachment efficiency even during long-term culture (10 passages). In addition, hiPSCs after 10 passages had normal karyotypes and still maintained an undifferentiated state and pluripotency. Thus, the safety of the RV method was confirmed. In the case of heart regenerative therapy, the number of cells required is on the order of 10^9^ per person. Considering this, the RV method will greatly aid the mass culture required for such therapies. Furthermore, its improvements in culture efficiency will have great impact in the repetitive process of subculture.

In the future, as tissue engineering and regenerative medicine using autologous cultured cells develops, the time required to culture the necessary numbers of cells will become more and more important. In such an era of cell therapy, the RV cell detachment method will be an essential technique for mass cultivation in large vessels with multilayered substrates. Furthermore, this method can easily be applied in automated cell culture systems using 2D culturing, since the method requires no human hands.

## Methods

### Measurement of vibration characteristics

The vibration characteristics of SCP (CellStack^®^ Culture Chamber; Corning, NY, USA) were measured using a laser Doppler vibrometer (LV-188; Ono Sokki, Yokohama, Japan) with an input voltage to the vibration generator (F-60K/60, EMIC Corporation, Tokyo, Japan) of 35 V_p-p_. The amplitude of vibration velocity, *A*_*v*_, was measured at the centers of cultivation layers and at the jig, and then converted to vibration amplitude, *A*, by

1 $$A={A}_{v}/2\pi f.$$

Additionally, the relative amplitude of substrate against that of the jig was calculated. Note that the input power is constant at the vibration generator, i.e., the amplitude of the vibration generator decreases as frequency increases. The resonance frequency was identified from the relative amplitude, *A*_*r*_, calculated as,

2 $${A}_{r}={A}_{l}/{A}_{j},$$

where, *A*_l_ is the amplitude of the target layer of SCP, and *A*_j_ is the amplitude of the jig fixed to the vibration generator. When measuring amplitude distribution, vibration amplitudes were measured along the longer central axis of the cultivation layers. The input voltage to the vibration generator was set to 35 V_p-p_, and the driven frequency was adjusted to each resonance. Furthermore, the relative amplitude at each point was normalized by the maximum value.

### Cell preparation

C2C12 mouse myoblast cells (RCB0987; Riken Bio Resource Center, Ibaraki, Japan), and hiPSCs (253G4; Kyoto University, Kyoto, Japan) were employed to evaluate the proposed RV method. The myoblasts were cultured in growth medium (D-MEM/F12 supplemented with 10% fetal bovine serum (FBS)), and hiPSCs were cultured in modified StemFit Medium^22^. Both were cultured in a 5% CO_2_ humidified atmosphere incubator at 37 °C. Cell passage was performed by trypsinization in 0.050% trypsin-EDTA (25300; Invitrogen, Carlsbad, CA, USA) for myoblasts and TryPLE Select (1X) (Thermo Fisher Scientific, Waltham, MA, USA) for hiPSCs with pipetting.

Both cell types were passaged the minimum number of times needed for proliferation and were seeded in SCPs. In the experiments employing myoblasts, 3.5 × 10^6^ cells in 100 mL growth medium were seeded into each chamber of an SCP, i.e., 3.5 × 10^6^ cells in 100 mL and 1.75 × 10^7^ cells in 500 mL were seeded into single-layer and 5-layer SCPs, respectively. In the case of hiPSCs, we seeded 4 × 10^7^ cells into a 5-layer SCP coated with Matrigel (BD Biosciences, Franklin Lakes, NJ, USA). Cells at passage 4 were seeded with 100 mL growth medium into a 5-layer SCP coated with Matrigel. Both cell types were incubated for 4 days in a 5% CO_2_ humidified atmosphere incubator at 37 °C. The medium was exchanged 2 days after seeding. The cells were used as an initial sample in the following cell detachment experiments.

### Cell detachment methods

To prove that the RV method is effective in detaching cells from the cultivation substrates of SCPs, we detached cells using both trypsinization with resonance vibrations in the RV method and trypsinization with manual tapping in the CE method.

In both methods, cells attached to the cultivation substrates were washed twice with phosphate-buffered saline (PBS), and then PBS (50 mL in each chamber) was spread into the chamber. Myoblasts and hiPSCs were detached from the substrate by the following step:

1. RV method: 0.0125% trypsin-EDTA for myoblasts or 50% TryPLE Select (1X) for hiPSCs was spread into the SCP (30 mL in each layer). After 2 min, the RVs were excited for 1 min in the case of myoblasts, i.e., the total duration of treatment was 3 min. For the hiPSCs, after 2 min, the resonance vibrations were excited for 1 min, and the SCP was allowed to rest for 2 min, i.e., the total duration of treatment was 5 min.
2. CE method: 0.0125% trypsin-EDTA for myoblasts or 50% TryPLE Select (1X) for hiPSCs was spread onto the SCP (30 mL in each layer). After 2 min, manual tapping was applied 3 times on each side of the SCP for 1 min in the case of myoblasts, i.e., the total duration of treatment was 3 min. In the case of hiPSCs, after 2 min, manual tapping was applied 3 times on each side of the SCP for 1 min, and the SCP was allowed to rest for 2 min, i.e., the total duration of treatment was 5 min. In order to reduce human error/intention, the technicians, who routinely culture hiPS cells using SCP^22^ and do not know the purpose of the research, curried out the experiments.

Note that, we used the half diluted trypsin-EDTA and TryPLE Select compared to standard concentration. This is because that trypsin-EDTA at a high concentration is known to damage cells^19,27^ and TryPLE Select is recommended to be diluted half in the protocol of the Center for iPS Cell Research and Application (CiRA) at Kyoto University. After the detachment process, medium was spread onto the SCP (15 mL in each layer) to neutralize trypsin. Cells were collected from the cultivation chamber by pipetting. In this procedure, to completely collect the detached cells, each cultivation substrate was washed with PBS (30 mL per layer) twice. In addition, the sweep frequency ranges of the AC input to the vibration generator were 50–250 Hz for a single-layer SCP and 50–300 Hz for a 5-layer SCP, which covered the range of resonance frequency up to the third out-of-plane resonance. The input voltage was set to 35 V_p-p_.

### Counting of detached cells

The number of collected cells was counted using a hemocytometer (A116; Asone, Osaka, Japan) under a phase contrast microscopy (ECLIPSE Ti; Nikon Corporation, Tokyo, Japan), and this process was repeated 4 times. From this result, the ratio of detached cells to total cells (collected cells plus remaining cells) was calculated. Additionally, the ratio of cell survival was measured with the Trypan blue (Trypan Blue Solution (0.4%); Thermo Fisher Scientific) exclusion test on a hemocytometer, because cells may have died during the detachment process.

### Counting of remaining cells

After detachment, the cells remaining in the SCP were removed from the cultivation substrates using thick trypsin-EDTA for a prolonged time and counted. That is, the cells were soaked in 0.025% trypsin-EDTA (15 mL in each layer) for 10 min at 37 °C, and then collected by pipetting. In addition, to completely collect the cells, the cultivation substrates were washed with PBS (30 mL in each layer) twice, and cells were collected in PBS.

### Cell proliferation assay

The cell proliferation ratio after the detachment process was evaluated by counting cell numbers after culture for a certain period of time. In particular, the myoblasts (8.0 × 10^4^ cells) detached with the RV and the CE methods were independently reseeded in 60 mm dishes (AGC Techno Glass Co., Ltd., Shizuoka, Japan) and incubated for 72 h. Afterwards, the number of cells was counted using a hemocytometer, and the cell counting was repeated 4 times.

### Immunofluorescence staining

To confirm the pluripotency of hiPSCs, the cells were immunostained with pluripotency markers. Cells were washed with PBS and fixed with 4% paraformaldehyde for 20 min at room temperature. They were then permeabilized with 0.1% Triton X-100 for 5 min at 37 °C in a CO_2_ incubator, and subsequently incubated with Immunoblock (KN001; DS Pharma Biomedical, Osaka, Japan) buffer for 30 min. Fixed cells were incubated with primary antibodies for 2 h. The primary antibodies used were as follows: 1:200 dilution of rabbit anti-NANOG (RCAB003P; ReproCELL, Yokohama, Japan), 1:200 dilution of mouse anti-OCT4 (sc-5279; Santa Cruz Biotechnology, Dallas, TX, USA), 1:200 dilution of anti-TRA1–60 (MAB4360; EMD Millipore, Billerica, MA, USA), and 1:200 dilution of mouse anti-SSEA4 (MAB4304; EMD Millipore). The cells were then incubated with secondary antibodies (Alexa Fluor 488/594 anti-mouse IgG or IgM and Alexa Fluor 488 anti-rabbit IgG) for 1 h at room temperature. After nuclear staining with Hoechst 33342, the cells were observed by fluorescence microscopy (Axio Observer; Carl Zeiss, Oberkochen, Germany).

### Long-term cell culture

Since the F-60K/60 is overengineered for vibrating of the SCP and this vibrator requires complicated operations, an optimal miniaturized vibration generator with the same conditions (50 to 250 Hz) was fabricated for long-term cell culture. The system as shown in Supplementary Fig. 2 is composed of an SCP and a vibration generator. The SCP was mounted on the vibration generator and fixed by toggle clamps. A swept frequency input signal was applied to the vibration generator using a function generator (AFG-2105; TEXIO TECHNOLOGY CORPORATION, Yokohama, Japan) and an amplifier (MA1-CE; IMV Corporation, Yamanashi, Japan). hiPSCs (253G4; Kyoto University, Kyoto, Japan) were employed to evaluate of 10 passages using the RV method. hiPSCs were cultured in modified StemFit Medium^22^ in a 5% CO_2_ humidified atmosphere incubator at 37 °C. As a control (CE), cell passage was performed by TryPLE Select (1X) (Thermo Fisher Scientific, Waltham, MA, USA) for hiPSCs with tapping. In the culture conditions of hiPSCs, we seeded 4 × 10^7^ cells into a 5-layer SCP coated with iMatrix-511^TM^ (Nippi, Inc.). Cells at passage 4 were seeded with 100 mL growth medium into a 5-layer SCP coated with iMatrix-511^TM^. The hiPSCs were incubated for 3–4 days in a 5% CO_2_ humidified atmosphere incubator at 37 °C. The medium was exchanged 2 days after seeding. The cells were passaged 10 times and we detached cells in the same conditions as described in “Cell detachment method”.

We show the growth curve of hiPSCs during the 10 passages, immunofluorescence staining images, FCA and karyotypes with the RV/CE methods. As shown in Fig. 5a, the total cell number, *N*_total_, in growth curve was calculated by,

3 $${N}_{total}={N}_{before}\cdot {N}_{detach}/{N}_{reseed}$$

where, *N*_before_, *N*_detach_ and *N*_seed_ are number of cells detached before a passage, number of detached cells and number of reseeded cells, respectively. Karyotypes of hiPSCs (Fig. 5b) were also analyzed by Nihon Gene Research Laboratories, Inc. (Sendai,16 Japan, http://www.ngrljapan.com). We conducted FCA of SSEA4 and TRA1-60, which are well-known surface markers of hiPSCs, after 10 passages. FCA was conducted on cells detached by both methods (Fig. 5c,d), reseeded, and cultured for 72 h. The cells subcultured by RV and CE were immunostained with pluripotency markers; NANOG (red), OCT4 (green), TRA1-60 (green), SSEA4 (red), and nuclei (blue) as shown in Supplementary Fig. 3a,b. For the immunofluorescence staining process, “immunofluorescence staining” in Methods was referred. Additionally, ectoderm, mesoderm, and endoderm differentiated from hiPCs passaged by RV method were observed for confirmation of pluripotency. The cells differentiated from hiPSCs passaged 10 times by RV and CE were immunostained with ectoderm, mesoderm, and endoderm markers; BrachyuryT (green), SOX17 (green), βIII-tubulin (green), respectively, and nuclei (blue) as shown in Fig. 5e.

### Differentiation of hiPSCs into ectoderm, mesoderm, and endoderm

To induce neural stem cells or definitive endoderm derived from hiPSCs, we used STEM Definitive Endoderm Kit (STEMCELL #05110) according to the manufacturer’s instructions. In neural cells, we induced spontaneously with MEMα plus 5% FBS. To induce mesoderm, we used RPMI (Wako) with B27 minus insulin (Life Technologies) and CHIR99021 (Wako) from day 0 to day 1, as described previously^31^. Cells were fixed and used for immunostaining on day 4, 7, 3, respectively.

### Measurement of flow cytometry analysis (FCA)

The cells (1.0 × 10^6^ cells) were suspended in 100 µL PBS with 2% FBS, and each antibody was reacted at an appropriate concentration, i.e., 1:10 dilution of isotype control (130-104-612; Miltenyi, Bergisch Gladbach, Germany), 1:10 dilution of anti-SSEA-4-PE antibodies (clone: REA101; 130-100-635; Miltenyi), and 1:10 dilution of anti-TRA-1-60-PE antibodies (clone: REA157; 130-100-350; Miltenyi), for 30 min on ice. The antibodies were washed with PBS, and the cells were then suspended in 1 mL PBS with 2% FBS, analyzed using a Gallios Flow Cytometer (Beckman Coulter, Brea, CA, USA), and analyzed using Kaluza software (Beckman Coulter).

### Statistics

Values are presented as means ± SDs. Statistical significance was evaluated using Student’s t tests for comparisons between two mean values. Results with *p* values of less than 0.05 were considered significant.

## Supplementary information

Supplementary Information
