# Supplementary material for: An effective detachment system for human induced pluripotent stem cells cultured on multilayered cultivation substrates using resonance vibrations
Source: Sci Rep. 2019 Oct 30;9:15655. doi: 10.1038/s41598-019-51944-w (PMC6821886; doi:10.1038/s41598-019-51944-w)
Supplement: Supplementary file 1 — Supplementary Information [file 41598_2019_51944_MOESM1_ESM.docx]

**Supplementary Information**

**An effective detachment system for human induced pluripotent stem cells cultured on multilayered cultivation substrates using resonance vibrations**

Yusuke Terao^1,*^, Yuta Kurashina^2,3,*^, Shugo Tohyama^4,5,*^, Yuki Fukuma^1^, Keiichi Fukuda^4^, Jun Fujita^4,**^, Kenjiro Takemura^2,**^

^1^School of Science for Open and Environmental Systems, Graduate School of Science and Technology, Keio University, 3-14-1 Hiyoshi, Kohoku-ku, Yokohama, 223-8522, Japan

^2^Department of Mechanical Engineering, Faculty of Science and Technology, Keio University, 3-14-1 Hiyoshi, Kohoku-ku, Yokohama, 223-8522, Japan

^3^Department of Materials Science and Engineering, School of Materials and Chemical, Tokyo Institute of Technology, Technology, 4259 Nagatsutacho, Midori-ku, Yokohama, 226-8503, Japan

^4^Department of Cardiology, Keio University School of Medicine, 35 Shinanomachi Shinjuku-ku, Tokyo 160-8582, Japan

^5^Department of Organ Fabrication, Keio University School of Medicine, 35 Shinanomachi, Shinjuku-ku, Tokyo 160-8582, Japan

^*^These authors contributed equally to the paper.

^**^Contact: jfujita@keio.jp and takemura@mech.keio.ac.jp

Short running title: Highly efficient detachment of human induced PSCs

**Supplementary Note**

We calculated the impact on the SCP from both hand slapping and resonance vibration. As shown in Figures 3 and 4, the RV method detaches cells more effectively than does the CE method. This may be because of differences in force applied to the cells by each method. In the CE method, the momentum conservation can be expressed as

$m_{\mathrm{hand}}\cdot v_{\mathrm{hand}}= m_{\mathrm{SCP}}\cdot v_{\mathrm{SCP}}$ (S1)

where, *m* and *v* represent mass and velocity, respectively. Note that subscripts denote hand and SCP. The acceleration of slapped SCP, *a*_SCP_, can be expressed with contact time, *Δt*, of hand and SCP as

$a_{\mathrm{SCP}}= \frac{v_{\mathrm{SCP}}}{\Delta t}$. (S2)

Since cells attached to a substrate layer of an SCP are subjected to the same acceleration as is the SCP, the force, *F*_CE_, applied to the cells by the CE method is assumed to be

$F_{\mathrm{CE}}= m_{\mathrm{cell}}\cdot a_{\mathrm{cell}}= \frac{m_{\mathrm{cell}}{\cdot m}_{\mathrm{hand}}{\cdot v}_{\mathrm{hand}}}{m_{\mathrm{SCP}}\cdot\Delta t}$. (S3)

The mass of the 5-layer SCP including medium was 0.93 kg. Assuming that *m*_hand_ = 0.49 kg (mass ratio of the hand in the human body (60 kg) is about 0.8 %), *m*_cell_ = 8.0 × 10–15 kg (average weight of all cell types), *v*_hand_ = 0.5–1.0 m/s, and *Δt* = 0.5 s, the force applied to the cells can be estimated as

$F_{\mathrm{CE}}=2.1 \times{10}^{-14} \sim4.2 \times{10}^{-14} N$. (S4)

On the other hand, in the RV method, the exciting force, *F*_RV_, applied to the cells can be calculated with the vibration frequency, *f*, and the amplitude, *A*_sub_, by

$F_{\mathrm{RV}} = m_{\mathrm{cell}} \cdot a_{\mathrm{sub}}= m_{\mathrm{cell}}\cdot\frac{A_{\mathrm{sub}}}{2} \cdot\left( 2\pi f \right)^{2}$. (S5)

In the cell detaching experiment using the 5-layer SCP, the driving frequency was 50–300 Hz (see METHODS) and the vibration amplitude on the substrates is 0.5–3.0 mm (see Figure 3A). Substituting these values into Eq. 5, we have

$F_{\mathrm{RV}}= 2.0 \times{10}^{-13} \sim4.3 \times{10}^{-11} N$. (S6)

Consequently, the impact force by the CE method is 2.1 × 10^-14^–4.2 ×10^-14^ N, and the impact force by the RV method is 2.0 × 10^-13^–4.3 ×10^-11^.

**References**

Tohyama S., Fujita J., Fujita C., Yamaguchi M., Kanaami S., Ohno R., … Fukuda K. (2017). Efficient large-scale 2D culture system for human induced pluripotent stem cells and differentiated cardiomyocytes. *Stem Cell Reports*. *9*, 1406-1414. doi: 10.1016/j.stemcr.2017.08.025

**
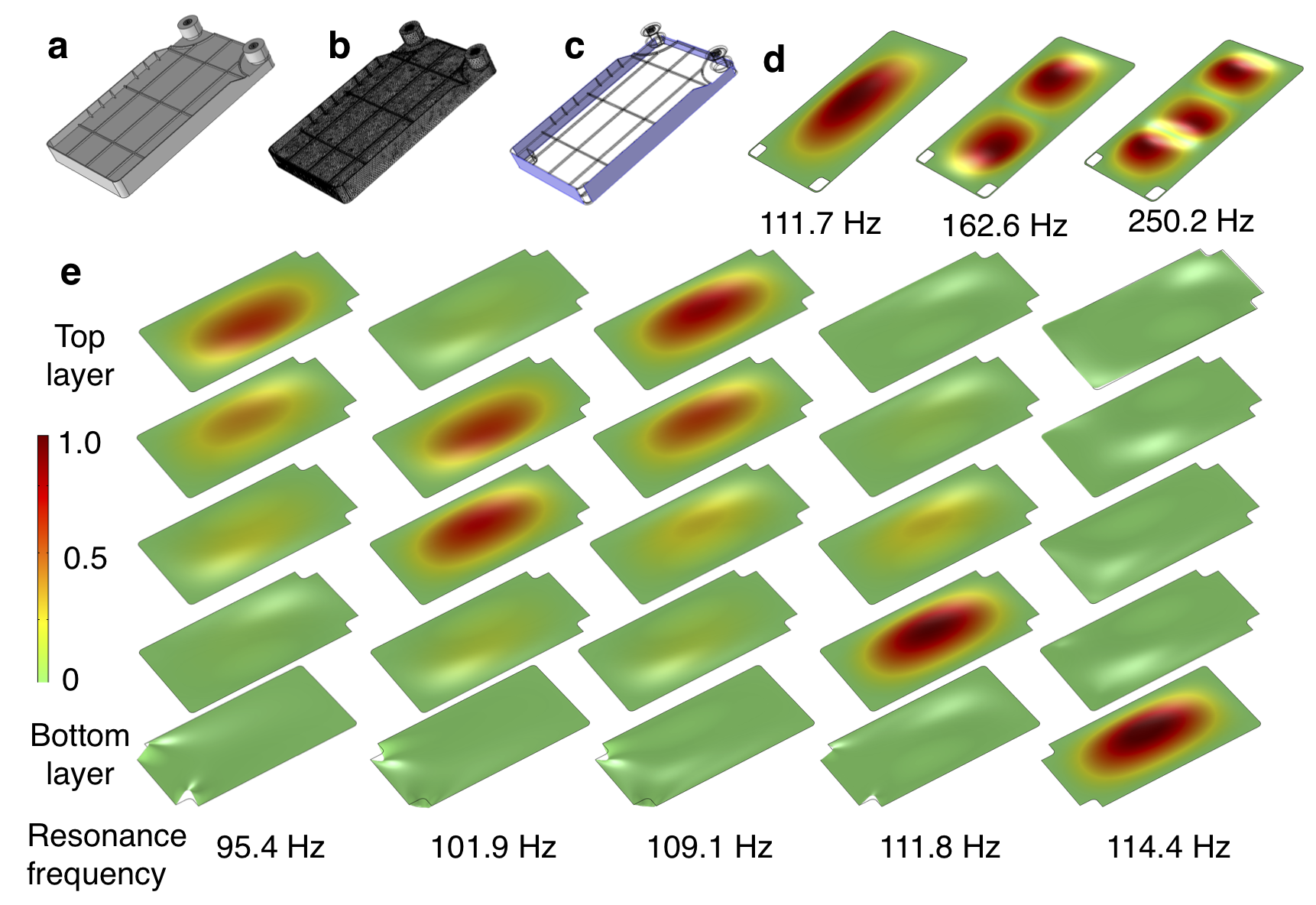
**

**Figure S1 | Three-dimensional (3D) CAD model of SCP with a single layer used for eigenvalue analysis and results of eigenvalue analysis.** **(a)** 3D CAD model, **(b)** 3D mesh model using tetrahedral elements; **(c)** side planes colored in purple are fixed for eigenvalue analysis. **(d)** Eigenvalue analysis results for the SCP. The color bar indicates the normalized displacement in the out-of-plane direction by the maximum value. The first out-of-plane vibration mode was at 111.7 Hz, the second at 162.6 Hz, and the third at 250.2 Hz. **E**: Eigenvalue analysis of the first out-of-plane mode of 5-layered SCP. The color bar indicates the normalized displacement in the out-of-plane direction by the maximum value. Resonance frequency ranges between 95.6 and 114.4 Hz.

**Figure S2 | Representative immunofluorescence staining for NANOG (red), OCT4 (green), TRA1-60 (green), SSEA4 (red), and nuclei (blue).** hiPSCs were stained after detachment by **(a)** the RV and **(b)** the CE methods 10 times repeatedly. Scale bars represent 100 µm.

**Figure S3 | Differentiation of hiPSCs into ectoderm, mesoderm, and endoderm.** Immunofluorescence staining of ectoderm, mesoderm, and endoderm for BrachyuryT (green), SOX17 (green), βIII-tubulin (green), and nuclei (blue) in hiPSC-derived dispersed cells after 10 passages with the RV and the CE methods, respectively. Scale bars represent 100 µm.

**Table SI | Resonance frequency of each layer of the 5-layer SCP.**
